# High frequency stimulation induces sonic hedgehog release from hippocampal neurons

**DOI:** 10.1038/srep43865

**Published:** 2017-03-06

**Authors:** Yujuan Su, Yuan Yuan, Shengjie Feng, Shaorong Ma, Yizheng Wang

**Affiliations:** 1 Laboratory of Neural Signal Transduction, Institute of Neuroscience and State Key Laboratory of Neuroscience, Shanghai Institutes for Biological Sciences, Chinese Academy of Sciences, University of Chinese Academy of Sciences, Shanghai, China; 2 Laboratory of Neural Signal Transduction, Institute of Neuroscience and State Key Laboratory of Neuroscience, Shanghai Institutes for Biological Sciences, Chinese Academy of Sciences, Shanghai, China.

## Abstract

Sonic hedgehog (SHH) as a secreted protein is important for neuronal development in the central nervous system (CNS). However, the mechanism about SHH release remains largely unknown. Here, we showed that SHH was expressed mainly in the synaptic vesicles of hippocampus in both young postnatal and adult rats. High, but not low, frequency stimulation, induces SHH release from the neurons. Moreover, removal of extracellular Ca^2+^, application of tetrodotoxin (TTX), an inhibitor of voltage-dependent sodium channels, or downregulation of soluble n-ethylmaleimide-sensitive fusion protein attachment protein receptors (SNAREs) proteins, all blocked SHH release from the neurons in response to HFS. Our findings suggest a novel mechanism to control SHH release from the hippocampal neurons.

SHH is a secreted protein and functions as a mitogen or morphogen in embryonic development of the CNS^1^. In the producing cells, after the signal peptide of a full length SHH is cleaved, the resulting N-terminal signaling domain is then cholesterolated and palmitylated to be functional before secreted. In the receiving cells, the N-terminal SHH binds to Patched1 (PTCH1) to relieve PTCH1 inhibition on Smoothened (SMO), a transmembrane protein homologous with members of G-protein coupled receptors, to trigger downstream Gli1 transcription and affect various biological processes^2^,^3^,^4^.

Though SHH is best known as a key regulator during embryonic development, it has an important role in adult tissue homeostasis^5^. For example, it plays important roles in the regulation of adult neural progenitor cell proliferation as well as in the formation of dendritic spines^6^. However, little is known about SHH release in the neurons after their fate is determined.

The PTCH1 and SMO have been reported to localize at the synapse of the postnatal and adult hippocampus^7^. Moreover, it has been reported that enhancing intracellular Ca^2+^ can induce SHH release in a gastric acid secretion model^8^,^9^. Further, exposure to high potassium can increase the amount of SHH protein in the medium of cultured PC6 cells^10^. Increase in intracellular Ca^2+^ or extracellular potassium can stimulate cell excitation. In ischemia and temporal lobe epilepsy, SHH expression is specifically increased in neurons, but not in astrocytes^11^. Additionally, SHH is quickly released under epileptic, but not physiological conditions. The released SHH can rapidly regulate extracellular glutamate levels and affect the development of epilepsy^12^.

In the current study, after confirming the synaptic localization of SHH in the young postnatal and adult hippocampus by synaptosome fractionation, vesicle isolation and immunoelectron microscopy studies, we used cultured hippocampal neurons and acute hippocampal slices to explore whether increase in neuronal activity by electrical stimulation can induce SHH release. We found that electrical stimulation at 100 Hz, but not at 10 Hz, can induce SHH release specifically from the neurons, but not from the astrocytes, in a manner that depends on extracellular Ca^2+^ and SNAREs proteins.

## Results

### Expression and localization of SHH

**Figure 1:**
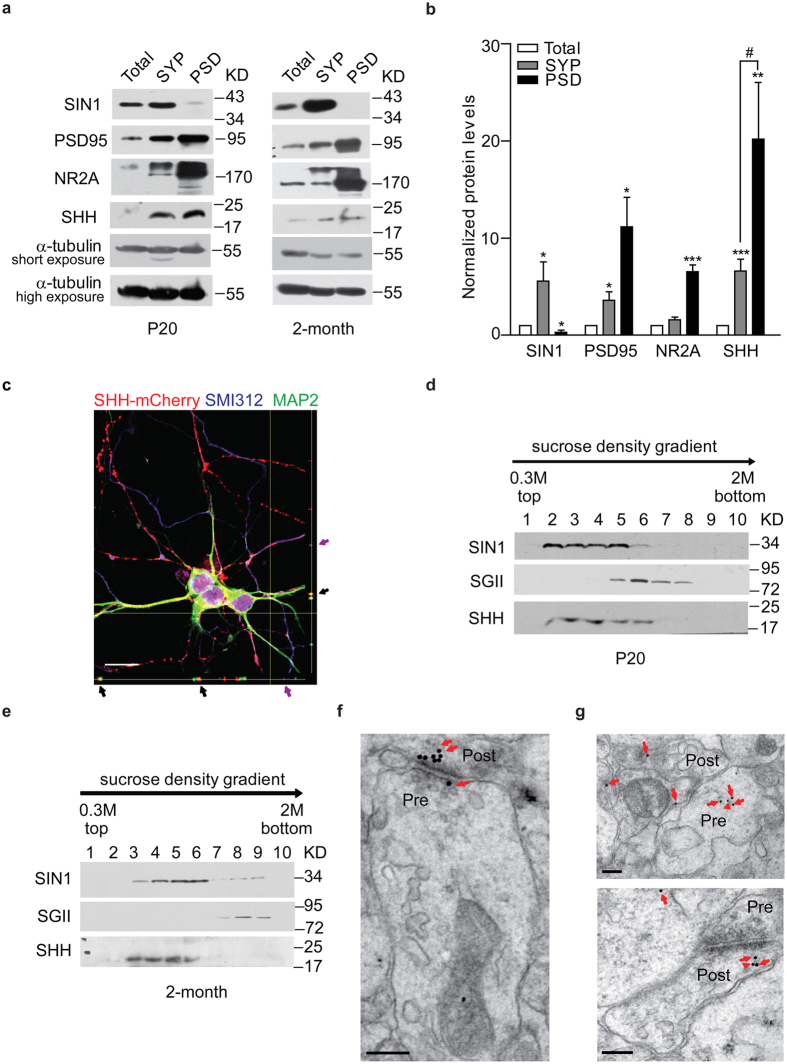
Expression and localization of SHH. (**a**) Representative immunoblots of SHH in the total lysate (Total), synaptosome (SYP) and post-synaptic density (PSD) fractions of P20 or 2-month-old rat hippocampus. α-tubulin: loading control. (**b**) Quantification of the protein levels in (**a**). For SIN1, N = 5, P = 0.048 for SYP vs. Total, P = 0.015 for PSD vs. Total. For PSD95, N = 4, P = 0.029 for SYP vs. Total, P = 0.016 for PSD vs. Total. For NR2A, N = 4, P = 0.081 for SYP vs. Total, P = 0.0002 for PSD vs. Total. For SHH, N = 6, P = 0.0009 for SYP vs. Total, P = 0.008 for PSD vs. Total, P = 0.047 for PSD vs. SYP. Paired t-test was used. Data were means ± s.e.m. *P < 0.05, **P < 0.01, ***P < 0.001 vs. Total. ^#^P < 0.05 vs. SYP. (**c**) Immunostaining of cultured hippocampal neurons expressing SHH-mCherry with antibodies against SMI312 (the axon marker) and MAP2 (the dendritic marker). The co-localization of SHH with SMI312 (purple) or MAP2 (black) shown in the orthogonal view are marked by arrows. (**d** and **e**) Representative immunoblots of SHH mainly in SIN1-labeled synaptic vesicles (SVs), but mildly in secretogranin II (SGII)-labeled large dense core vesicles (LDCVs) at P20 (**d**) or 2-month-old (**e**) rat hippocampus (N = 4). (**f**) Representative electron microscopic image of immunogold labeling by the antibody against SHH (red arrows) showing its localization in pre-synaptic terminals (pre) and post-synaptic soma (post) of P20 rat hippocampus. (**g**) Electron microscopic images of P20 rat hippocampus showing SHH vesicle localization in pre-synaptic terminals (pre) using the antibody against SHH (red arrowheads) with the antibody against SIN1 (red arrows, up), and in post-synaptic soma (post) with the antibody against SGII (red arrows, down). Scale bars: 20 μm for (**c**), 0.2 μm for (**f** and **g**). The full-length gels of immunoblots in (**a**) are in Supplementary Fig. S1, in (**d** and **e**) are in Supplementary Fig. S3a and b.

We initially examined whether SHH is expressed in the synapse of rat hippocampus at the age of postnatal 20 days (P20) or 2 months old (2-month), on behalf of young postnatal and adult animals, a method similar with previous reports^7^. Following the reported protocols^13^,^14^, we performed fractionation experiments followed by immunoblot analysis to study SHH expression in synaptosome (SYP) or post-synaptic density (PSD) of the hippocampus from P20 and 2-month-old rats. Representative immunoblots were shown in Fig. 1a and Supplementary Fig. S1. Analysis of the band densities revealed that SHH expression was enriched in the SYP and PSD fractionations when compared with that in the total lysates (Total) (P = 0.0009 for SYP vs. total, P = 0.008 for PSD vs. total). Further, the level of SHH was 3-fold higher in the PSD fractionation than that in the SYP fractionation (P = 0.047 for PSD vs. SYP) (Fig. 1b). These results suggest that SHH is present at both pre-synaptic and post-synaptic sites of the hippocampal neurons, but mainly at post-synaptic sites. To provide additional evidence to support SHH post-synaptic localization, we transfected cultured hippocampal neurons with the lowest level of mCherry-tagged SHH (SHH-mCherry) and examined its localization. As shown in Fig. 1c and Supplementary Fig. S2, mCherry fluorescence in the axon, the dendrite and the post-synapse was evident, further suggesting the synaptic localization of SHH proteins. To show whether SHH localize in the vesicles, we conducted the sucrose gradient centrifugation and examined its vesicle localization. As shown in Fig. 1d and Supplementary Fig. S3a, for P20 rat hippocampus, SHH proteins were more concentrated at synaptophysin1 (SIN1)-labeled synaptic vesicles (SVs) than secretogranin II (SGII)-labeled large dense-core vesicles (LDCVs). However, for 2-month-old rat hippocampus, SHH proteins were found mainly in SIN1-labeled SVs, but mildly in SGII-labeled LDCVs (Fig. 1e and Supplementary Fig. S3b). Our results thus suggest that SHH is present in the vesicles at both pre-synaptic and post-synaptic sites.

Then, we performed immunoelectron microscopy to further study SHH synaptic localization. As shown in Fig. 1f and Supplementary Fig. S4, using SHH antibody and the second antibody conjugated with gold particles, we found vesicular localization of SHH at both pre-synaptic and post-synaptic sites of the neurons in P20 rat hippocampus. The results are consistent with the findings in above fractionation studies and data in the previous report^15^. Moreover, in P20 rat hippocampus, using the primary antibody against SHH, the antibodies against SIN1 (Fig. 1g, up) or SGII (Fig. 1g, down), and the secondary antibodies conjugated with 10-nm or 18-nm gold particles, we found that SHH was co-localized with both SIN1-labeled SVs and SGII-labeled LDCVs. All together, these results suggest the vesicle localization of SHH at the synaptic zone of rat hippocampus.

### High, but not low, frequency stimulation induces SHH release from the neurons, but not from the astrocytes

The vesicle localization of SHH provides anatomical evidence to imply that the increase in neuronal activity might trigger its release. We then explored whether the increase in neuronal activity by field electrical stimulation, a method widely used to induce brain-derived neurotrophic factor (BDNF) release^16^,^17^, can induce SHH release from cultured hippocampal neurons and acute hippocampal slices. Bath-applied stimulation did evoke neuronal action potentials as assayed by whole-cell current clamp recording (Supplementary Fig. S5a and b). Moreover, treatment of the neurons with tetrodotoxin (TTX), an inhibitor of voltage-dependent sodium channels, suppressed the formation of action potentials (Supplementary Fig. S5c and d). Therefore, electrical stimulation does enhance neuronal activity. Further, we have confirmed that HFS at a maximal current intensity of 500 μA did not induce neuronal damage of hippocampal neurons (Supplementary Fig. S6a) or acute hippocampal slices (Supplementary Fig. S6b).

**Figure 2:**
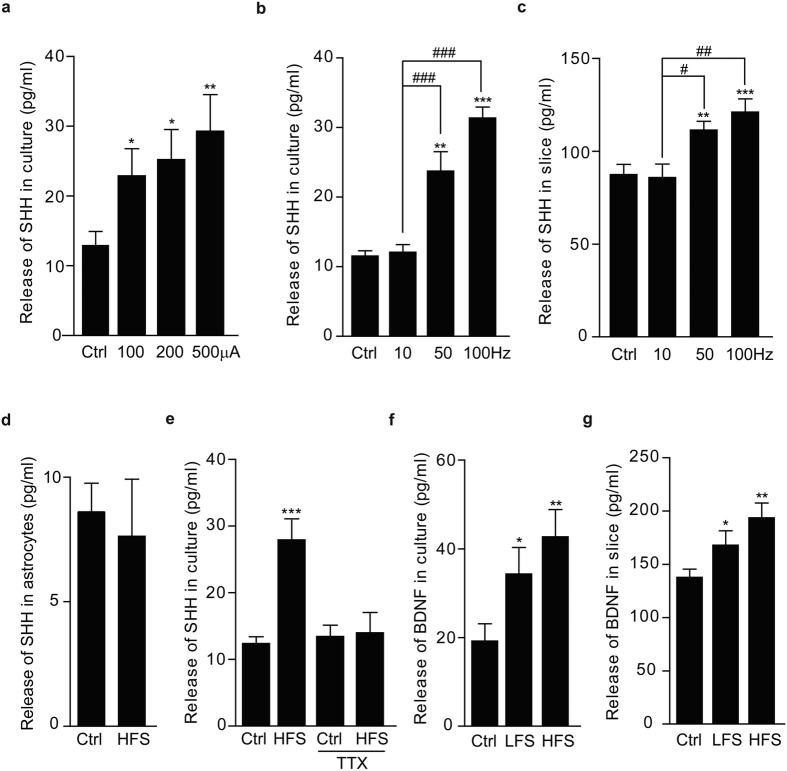
HFS, but not LFS, induces SHH release from the neurons. (**a**) Levels of SHH in the medium of cultured hippocampal neurons after stimulation with indicated current intensities. N = 7, P = 0.032 for 100 μA vs. Ctrl, P = 0.018 for 200 μA vs. Ctrl, P = 0.0097 for 500 μA vs. Ctrl, there is no significant difference among 100 μA, 200 μA and 500 μA groups. (**b** and **c**) Levels of SHH in the medium of cultured hippocampal neurons (**b**) or of acute hippocampal slices (**c**) after stimulation with indicated frequencies. (**b**) N = 5, P = 0.0035 for 50 Hz vs. Ctrl, N = 14, P = 6.93E-12 for 100 Hz vs. Ctrl, N = 5, P = 0.0002 for 50 Hz vs. 10 Hz, N = 9, P = 4.45E-08 for 100 Hz vs. 10 Hz. (**c**) N = 5, P = 0.002 for 50 Hz vs. Ctrl, N = 17, P = 0.0002 for 100 Hz vs. Ctrl, N = 5, P = 0.02 for 50 Hz vs. 10 Hz, N = 10, P = 0.005 for 100 Hz vs. 10 Hz. (**d**) Levels of SHH in the medium of cultured hippocampal astrocytes after HFS (500 μA) (N = 4). (**e**) Levels of SHH in the medium of cultured hippocampal neurons after HFS without or with TTX pretreatment (N = 6, F (1, 10) = 32.71, P = 0.0002 for HFS vs. Ctrl without TTX). (**f** and **g**) Levels of BDNF in the medium of cultured hippocampal neurons (**f**) or of acute hippocampal slices (**g**) after HFS or LFS. (**f**) N = 7, P = 0.048 for LFS vs. Ctrl, P = 0.0067 for HFS vs. Ctrl. (**g**) N = 10, P = 0.045 for LFS vs. Ctrl, P = 0.0019 for HFS vs. Ctrl. Control (Ctrl): no stimulation. HFS: 3 mins and 1 sec, 100 Hz, LFS: 3 mins and 10 secs, 10 Hz. Current intensity: 100 μA, for all experiments, unless stated. Paired t-test was used for (**a**–**d**, **f**,**g**). Two-way ANOVA was used for (**e**). Data were means ± s.e.m. *P < 0.05, **P < 0.01, ***P < 0.001. ^#^P < 0.05, ^#^P < 0.01, ^###^P < 0.001.

We found that HFS at 100 μA induced SHH release to a significant level and increase in the current intensity to 500 μA did not further enhance its release as assayed by enzyme-linked immuno sorbent assay (ELISA) (Fig. 2a). SHH levels in the medium of both cultured hippocampal neurons (Fig. 2b) and slices (Fig. 2c) were markedly enhanced in response to HFS at 50 Hz and 100 Hz, whereas low frequency stimulation (LFS) at 10 Hz, even for 30 minutes did not induce SHH release from the neurons (Supplementary Fig. S7a), suggesting that SHH release was high frequency-dependent. On the contrary, SHH level in the medium of cultured hippocampal astrocytes was not changed in response to HFS (Fig. 2d). Additionally, TTX abolished the HFS-induced SHH release (Fig. 2e), further suggesting the necessity of neuronal activity for SHH release. Moreover, the upregulation of transcription factor Gli1 has confirmed that SHH is indeed released upon HFS, and the secreted SHH then trigger its pathway activation (Supplementary Fig. S7b–d). Taken together, these results suggest that HFS did increase neuronal activities to induce SHH release from the neurons.

Further analysis revealed that the levels of BDNF, an important neuronal excitability-related neurotrophin^18^,^19^, in the medium of either cultured hippocampal neurons (Fig. 2f) or acute hippocampal slices (Fig. 2g) began to increase significantly even after LFS, the condition under which little SHH could be detected. There was little difference between the LFS- and HFS-induced BDNF release from hippocampal neurons and slices, suggesting that unlike SHH release, electrical stimulation-induced BDNF release was not high frequency- dependent.

### HFS induces SHH-pHluorin release from the neurons in real time

To provide further evidence that HFS indeed causes SHH release from the neurons, we examined the HFS effects on exocytosis of SHH through monitoring vesicle luminal pH changes using a more sensitive probe, the pH-sensitive GFP (pHluorin). The use of pHluorin for optical measurements of exocytosis has been well established and widely used^17^,^20^,^21^. The intensity of pHluorin fluorescence is greatly weakened as a result of the acid vesicle lumen, but increases rapidly during vesicle fusion since the pH of vesicle lumen changes from 5.2 to 7.4 as fusion occurs^20^,^21^.

We fused the N-terminal fragment of SHH, which has both lipid and palmityl modifications on its terminus and retains all known biological activities, with pHluorin to visualize its release upon electrical stimulation in real time. Using total internal reflection fluorescence (Tirf) microscopy, we are able to monitor the dynamic process showing SHH-pHluorin-containing vesicle fusion with the plasma membrane upon electrical stimulation. We expressed SHH-pHluorin in the human embryonic kidney 293 (HEK293) cells and confirmed its expression and release through western blot analysis (Supplementary Fig. S8a–d). Then, we expressed SHH-pHluorin in cultured hippocampal neurons on DIV7 and examined its release 3–7 days later using Tirf microscopy. To analyze fluorescence signals, neurons were co-transfected with SHH-pHluorin and mCherry, which can be used as a marker of neuronal morphology. For mCherry-positive neurons, axons and dendrites can be identified by neurite morphology. The electrical stimulation-induced SHH-pHluorin release could be examined by monitoring the fluorescence intensity changes of pHluorin.

Consistent with previous reports^20^,^21^, bath application of ammonium chloride (NH_4_Cl) solution, which deacidifies vesicular lumen, resulted in a marked increase in the fluorescence intensity at each SHH-pHluorin-containing punctum, while application of 2-N-morpholino ethane sulfonic acid (MES) solution had the opposite effect (Supplementary Fig. S9a). Free moving vesicles can also induce fluorescence intensity changes when they pass the observation field, but vesicles do not need ATPase in the absence of fusion. So we use bafilomycin A1, an inhibitor of the vesicular proton ATPase^22^, to distinguish those pass-by vesicles from release events. As Supplementary Fig. S9b and c showed, pretreatment with bafilomycin A1 could maintain a sustained fluorescence after SHH-pHluorin release through blocking vesicle lumen re-acidification when they fuse with the membrane. Together, we have confirmed the availability of using SHH-pHluorin system to monitor the SHH release upon electrical stimulation in real time.

**Figure 3:**
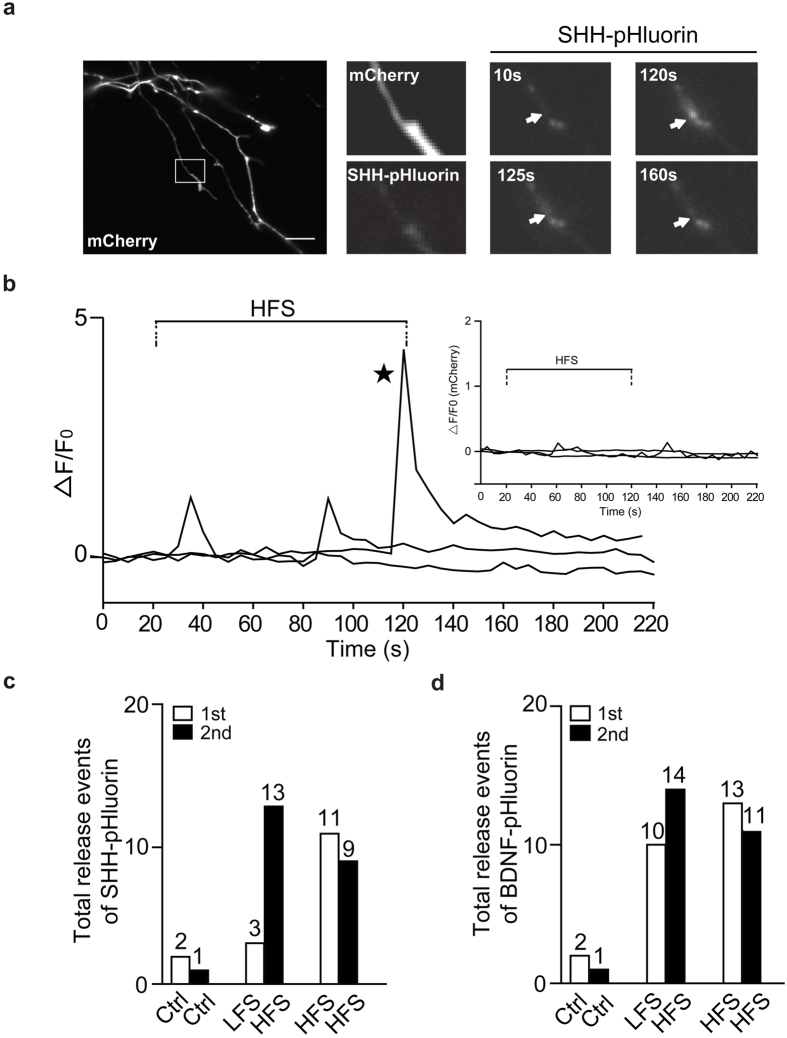
HFS induces SHH-pHluorin release from the neurons in real time. (**a**) (Left) Representative images (mCherry) of hippocampal neurons transfected with mCherry and SHH-pHluorin. Scale bar: 10 μm. Magnified views of SHH-pHluorin punctum in indicated boxed area (Left) at basal condition before stimulation (10 sec, Upper, Middle), or at indicated seconds during stimulation (120 sec, Upper, Right), or after stimulation (125 sec, 160 sec, Down). (**b**) Representative fluorescence changes of SHH-pHluorin at dendrites. The asterisk marks trace showing fluorescence changes of arrow-indicated punctum in (**a**, Right). Inset, corresponding fluorescence changes of mCherry. ΔF/F_0_, ΔF = Fx − F_0,_ F_0_, basal fluorescence intensity before stimulation, Fx, fluorescence intensities at indicated time x. HFS was applied from 20 to 120 secs for (**a** and **b**). (**c** and **d**) Release events of SHH-pHluorin (**c**) or of BDNF-pHluorin (**d**) after the first (1st) and second (2nd) stimulations. Numbers above columns are release events of six observation fields from at least three independent experiments.

We found that HFS tended to trigger SHH-pHluorin release in dendrites, with a stark, rapid rise and decline pattern of pHluorin fluorescence intensity (Fig. 3a and b), indicating fusion of SHH-containing vesicles upon stimulation. To study the LFS- and HFS-induced SHH-pHluorin release, for each observation field, we applied two sequential trials of stimulation for fluorescence intensity analysis. For the first trial of stimulation, we applied HFS to confirm the existence of SHH-pHluorin-containing vesicles in the observed field. For the second trial of stimulation, either HFS or LFS was applied to study the frequency dependence of SHH release. Compared with HFS, the fluorescence changes were more rarely found when LFS was applied. For statistic analysis, we collected all the release events of six observation fields from at least three independent experiments. As shown in Fig. 3c, the total number of release events from observation fields upon HFS was markedly larger than that upon LFS. Moreover, HFS tended to trigger SHH-pHluorin release from the dendrites as identified by neuronal morphology according to previous reports^23^,^24^,^25^ (Supplementary Fig. S9d), where SHH is found in the hippocampal neurons^15^. Consistent with the results above showing that LFS could induce BDNF release from the neurons, the total number of release events for BDNF-pHluorin was similar between LFS and HFS (Fig. 3d). Together, the ELISA and Tirf results both suggest that HFS, but not LFS, can induce SHH release from the hippocampal neurons.

### Both Ca^2+^ and SNAREs proteins are necessary for HFS-induced SHH release

**Figure 4:**
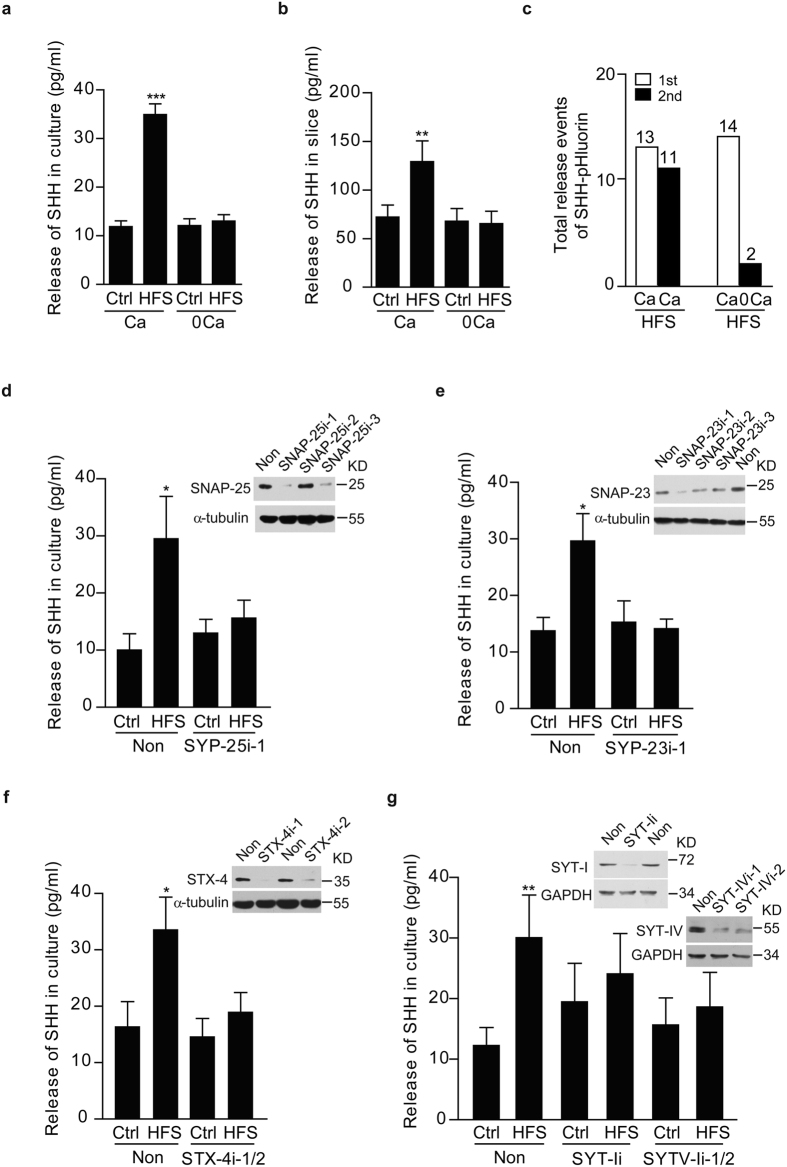
Ca^2+^ and SNAREs proteins are necessary for HFS-induced SHH release. (**a** and **b**) Levels of SHH released after HFS with (Ca) or without (0Ca) extracellular Ca^2+^ of cultured hippocampal neurons (**a**, N = 11, F (1, 20) = 81.54, P < 0.0001 for HFS vs. Ctrl with Ca) and of acute hippocampal slices (**b**, N = 12, F (1, 22) = 10.17, P = 0.0042 for HFS vs. Ctrl with Ca). (**c**) Release events of SHH-pHluorin after two trials of sequential HFS, with the second HFS in the presence (Ca) or absence (0Ca) of extracellular Ca^2+^. Numbers above columns are release events of six observation fields from at least three independent experiments. (**d**–**g**) Levels of SHH after HFS in the medium of cultured hippocampal neurons transfected with nonsense RNAi (Non) or RNAi against SNAP-25 (SNAP-25i-1, (**d**), N = 5, F (1, 6) = 11.51, P = 0.0146 for HFS vs. Ctrl with Non), SNAP-23 (SNAP-23i-1, (**e**), N = 5, F (1, 8) = 9.827, P = 0.0139 for HFS vs. Ctrl with Non), STX-4 (STX-4i-1/2, (**f**), N = 5, F (1, 8) = 10.47, P = 0.0120 for HFS vs. Ctrl with Non), SYT-I (SYT-Ii) or SYT-IV (SYT-IVi-1/2, (**g**), N = 8, F (2, 14) = 7.071, P = 0.0033 for HFS vs. Ctrl with Non). Insets, representative western blots for indicated proteins. α-tubulin and GAPDH: loading controls. Two-way ANOVA was used. Data were means ± s.e.m. *P < 0.05, **P < 0.01, ***P < 0.001 (**a**,**b**, **d**–**g**). The full-length gels of western blots in (**d**–**g**) are presented in Supplementary Fig. S11a–d.

It has been known that Ca^2+^ and SNAREs proteins are critical for neurotransmitter and neuropeptide release^26^,^27^. We then explored the role of Ca^2+^ in HFS-induced SHH release. Removal of Ca^2+^ from extracellular solution abolished HFS-induced SHH release from the neurons (Fig. 4a) and slices (Fig. 4b). The total number of HFS-induced SHH-pHluorin release was significantly decreased in the absence of Ca^2+^ (Fig. 4c), suggesting the necessity of extracellular Ca^2+^. Treatment of the neurons with nimodipine (Nimo), an agent known to bind specifically to postsynaptic L-type voltage-gated calcium channels (L-VGCCs) and block the Ca^2+^ entrance^28^, reduced HFS-induced SHH release (Supplementary Fig. S10). Therefore, HFS-induced SHH release from the neurons depended on Ca^2+^ entrance through L-VGCCs.

Vesicle exocytosis is facilitated by high-affinity interaction of a group of highly conserved proteins, called SNAREs proteins. Three core proteins: synaptobrevin, syntaxin (STX) and SNARE-associate protein (SNAP) form the SNAREs complex, which work together with an EF-hand-containing Ca^2+^ sensor protein synaptotagmin (SYT), and play a critical role in the sequential vesicle docking, priming, fusion and synchronization of neurotransmitter release. Since SNAREs proteins are important for Ca^2+^ -mediated vesicle release, we next asked whether knocking down its components before electrical stimulation could block the SHH release. As shown in Fig. 4d–g, Supplementary Figs S11a–d and S12a–e, downregulation of synaptosome-associated protein of 25kD (SNAP-25), localized primarily at pre-synaptic terminals^29^, or of synaptosome-associated protein of 23kD (SNAP-23), localized primarily at post-synapse^30^, or of syntaxin-4 (STX-4), a spine-specific protein^31^, blocked HFS-induced SHH release. In line with the necessity of Ca^2+^, downregulation of Ca^2+^ sensor protein synaptotagmin-I (SYT-I), localized primarily at pre-synaptic terminals^32^, or of synaptotagmin-IV (SYT-IV), localized primarily at post-synapse^33^, both inhibited SHH release. All together, these results suggest that SHH can be released specifically from the neurons upon HFS, but not LFS, in a manner that depends on Ca^2+^ and SNAREs proteins.

## Discussion

Among the many morphogenic signaling cascades in embryonic and adult development^18^,^34^,^35^, hedgehog signaling remains one of the least understood. Though SHH has been best known as a key regulator during embryonic development, components of SHH signaling pathway continue to express in the adult brain^5^,^36^,^37^. Its transcripts are found in GABAergic neurons located in various basal forebrain nuclei^38^. Moreover, the subcellular localization of SHH in primary neurons has been shown^15^. However, where SHH signaling takes place in the adult brain remains largely unknown. The preferential distribution of PTCH1 and SMO in the post-synapse of hippocampal neurons^7^ and our current results showing the synaptic localization of SHH in the postnatal and adult rat hippocampus, are consistent with the reported roles of SHH in axonal or dendritic growth^39^,^40^,^41^.

Although majority of SVs are located in axonal termini, SVs in the post-synapse has also been reported to participate in dendritic exocytosis^42^,^43^,^44^,^45^,^46^,^47^,^48^,^49^,^50^,^51^,^52^. Consistently, we found synaptophysin1 (SIN1)-labeled SVs in the post-synapse as assayed by immunoelectron microscopy (Supplementary Fig. S13). Using combination of different methods including cell fractionation, immunoelectron microscopy and Tirf imaging analysis, we have documented the vesicle localization of SHH in the post-synapse that can be released upon HFS.

It has been suggested that neuronal activity is necessary to influence SHH signaling in peripheral neurons^53^. Besides, the association between electrical activity and SHH during spinal cord development has been reported^54^,^55^. Using cultured hippocampal neurons and acute hippocampal slices, we showed that high neuronal activity can induce SHH release in an extracellular Ca^2+^ - and SNAREs proteins-dependent manner. Consistent with previous report^16^, bath-applied stimulation did evoke neuronal action potentials and TTX suppressed its formation as well as the HFS-induced SHH release, suggesting the necessity of neuronal activity for SHH release. With its continuous and specific expression in the adult CNS, the relationship between neuronal activity and SHH release in the neurons suggest that SHH might play a role in neurotransmission.

Moreover, BDNF, nerve growth factor (NGF) or neurotrophin-3 (NT-3) are reported to release in response to physiological stimulations^56^,^57^. In our study, both LFS and HFS induced a significant BDNF release from the neurons while SHH release is only HFS-sensitive. Downregulation of SNAP-25 blocked HFS-induced BDNF release from hippocampal neurons (Supplementary Fig. S14). We have thus suggested that in line with HFS-induced SHH release, BDNF release also depends on SNAREs proteins. In this context, it is possible that the HFS-dependent release of SHH might mediate certain specific actions initiated by conditions with a high neuronal activity, such as epilepsy and ischemia.

It is one of the important findings in our report that, in response to electrical stimulation, SHH is released specifically from the neurons, but not from the astrocytes. The detailed mechanism about SHH release is not entirely clear at the present. SNAREs proteins are necessary for glutamate release, it is thus likely that HFS induces glutamate release from pre-synaptic termini in an extracellular Ca^2+^-dependent manner. The abundant glutamate release or accumulation at the synaptic zone could further trigger SHH release, mainly from the post-synapse. Considering the fact that only HFS, but not LFS, induces SHH release from the neurons, it is thus possible that there might be sensory or gate-like proteins that control its release in response to stimulation at high frequency. The exploration of such proteins will explain why SHH release is strictly high neuronal activity-dependent, and might have implications to the understanding of excitatory toxicity-related disorders, such as ischemia and epilepsy. The novel finding that SHH is unique in its release and its relationship with neuronal activity in the CNS will expand our understanding of the diverse actions of hedgehog pathway.

## Methods

All experimental protocols using animals and cell samples were approved by Shanghai Institutes for Biological Science, Chinese Academy of Sciences.

### Synaptosome and post-synaptic density fractionation

SYP and PSD were prepared from sprague dawley (SD) rat hippocampus at the age of P20 or 2-month-old by sucrose gradient method as previously described^13^,^14^. All purification steps were performed at 4 °C. The protease inhibitors were added to all solutions. The hippocampus was rapidly isolated and homogenized in ice-cold buffer A (in mM: 320 sucrose, 4 Hepes, pH 7.4, for all experiments, unless stated). The homogenized extract was spun at 1,000 g for 10 minutes (mins) and the supernatant (S1) was saved. S1 was then centrifuged for 15 mins at 9,200 g to get the pellet (P1). P1 was then re-suspended and re-centrifuged for 15 mins at 10,500 g to collect the pellet (P2). The final P2 was suspended in 1 volume buffer B (in mM: 5 Tris-HCl, pH 7.4) and 9 volume of ice-cold H2O to osmotically lyse the SYP for 30 mins (20 up-and-down movements with a glass pipette during the process).

For preparation of SYP and PSD, we referred to the reported protocol^58^. The resulting lysate was loaded onto a discontinuous sucrose gradient (0.85 M/1 M/1.2 M sucrose solution in 6 mM Tris-HCl, pH 8.0), followed by centrifugation at 82,500 g for 2 hours (hrs). The fraction between 1 M and 1.2 M sucrose was collected and saved as the SYP fractions. The SYP fractions were further adjusted to buffer B and mixed with equal volumes of buffer C (in mM: 6 Tris, pH 8.1, and 1% Triton X-100). The suspension was spun at 32,800 g for 20 mins. The supernatant was discarded, and the resulting pellet was saved as the PSD fractions. In order to study the synaptic localization of SHH, the total lysate of hippocampus (Total), the SYP and the PSD fractions were adjusted to an equal protein concentration (5 μg/μl) using Bio-Rad Protein Assay and then processed for western blots. α-tubulin is the house-keeping protein. For statistic analysis in Fig. 1b, SYP and PSD were normalized with Total lysate. Six independent experiments were performed.

### Western blots

Total proteins extracted from cultured cells using sodium dodecyl sulfonate (SDS) lysis buffer (2% SDS, 10% glycerol, 0.1 mM dithiothreitol, and 0.2 M Tris-HCl, pH 6.8) were western-blotted with the indicated primary and secondary antibodies. Bound antibodies were detected by the enhanced chemiluminescence detection. The band optical densities were quantified by Image Quant software.

### Primary cell culture

Primary hippocampal neurons were isolated and cultured as described previously^59^. Briefly, the neurons were obtained by dissociating the hippocampus from SD rat brains at the age of embryonic day 18 (E18) and then seeded onto coverslips (No. 1 Glass, Warner Instruments, Connecticut, USA) coated with 50 μg/ml poly-D-lysine (PDL). Primary hippocampal astrocytes were isolated and cultured as described with some modifications^60^,^61^. Cells were cultured at a density of 2 × 10^4^/cm^2^ for immunohistochemistry study, of 2 × 10^5^/cm^2^ for ELISA study, of 3 × 10^4^/cm^2^ for Tirf microscopy, and of 7 × 10^4^/cm^2^ for western blots. All cells were cultured in neurobasal medium supplemented with B-27 and 0.5 mM glutamax for 10 days before use.

### Transfection

For ELISA studies, the primary hippocampal neurons were transfected with indicated oligo RNAi on DIV7 and were used 72 hrs later. For Tirf study, the primary hippocampal neurons were transfected with SHH-pHluorin and mCherry following the Ca^2+^-phosphate transfection protocol^62^ and were used 72 hrs later. The HEK293 cells were obtained from the Cell Bank of the Chinese Academy of Sciences. The cells cultured in dulbecco’s modified eagle medium (DMEM) supplemented with 10% fetal bovine serum (FBS) were transfected with 1 μg of indicated plasmids using lipofectamine 2000 for western blots. The SHH construct was from Dr. Yun Zhao and the pHluorin construct was from Dr. Hailan Hu, both in the Chinese Academy of Sciences.

### Immunohistochemistry

Hippocampal neurons were cultured at a density of 2 × 10^4^/cm^2^ in 8 mm slide for 10 days. Transfected neurons were firstly washed twice with phosphate buffer solution (PBS, 0.1 M, for all experiments, unless stated) and fixed in 4% paraformaldehyde (PFA) for 20 mins. The neurons were treated with 0.3% Triton X-100 for 10 mins at room temperature (RT), followed by sequential overnight incubations with indicated antibody at 4 °C and fluorescent secondary antibodies diluted in PBS containing 3% bovine serum albumin and 0.3% Triton X-100 for 2 hrs at RT. The fluorescent signals were examined using an A1R laser-scanning confocal microscopy (Nikon, Japan).

### Vesicles isolation

For isolation of vesicles, we referred to the reported protocol^5^. Immediately following the SYP preparation, we suspended it in sucrose buffer (in mM: 200 sucrose, 0.1 MgCl_2_, 0.5 EGTA, 10 Hepes) and laid it onto a continuous sucrose gradient ranging from 0.3–2.0 M sucrose in 4 mM Hepes, and the gradient was centrifuged at 100,000 g for 3 hrs. In order to study the vesicle localization of SHH, the centrifuged samples were processed for western blots with same loading volume and probed with corresponding antibodies. The synaptic vesicle (SV) marker synaptophysin1 (SIN1, Synaptic System, Germany, Cat. No. 101011, 1:1000 dilution) and large dense core vesicle (LDCV) marker secretograninII (SGII, Santa Cruz, USA, Cat. No. SC-50290, 1:1000 dilution) were used to confirm the success isolation of vesicles. Thus, the distribution of SHH in specific vesicles with various densities can be studied. Four independent experiments were performed.

### Immunolabeling and electron microscopy

Immunogold electron microscopy was performed by post fixation immunogold labeling as described^63^. Briefly, hippocampal sections (20 μm) of SD rats at the age of P20 were embedded in epon-spurr resin. Thin sections (30–40 nm) were collected on nickel mesh grids. To study the synaptic localization of SHH, we used the antibody against SHH (Cell Signaling, USA, Cat. No. 2287 S, 1:100 dilution) and the secondary rabbit antibody conjugated to 18-nm gold particles (Sigma Aldrich, USA, 1:2000 dilution) (Fig. 1f and Supplementary Fig. S4). To study the vesicle localization of SHH, we used the antibody against SHH with the antibody against SIN1 (Synaptic System, Germany, Cat. No. 101011, 1:100 dilution), or with the antibody against SGII (Santa Cruz, USA, Cat. No. SC-50290, 1:100 dilution), respectively. The secondary rabbit or mouse antibodies conjugated to 10-nm or 18-nm gold particles (Sigma Aldrich, USA, 1:2000 dilution) were then applied (Fig. 1g). Two independent experiments were performed. For each experiment, we photographed twenty-five randomly selected fields for analysis.

### Electrical stimulation

Electrical stimulation was performed via two parallel tungsten wires. Current intensity was controlled by a constant current isolated stimulator and stimulation protocols were synchronized by Master-8 (A.M.P.I., Israel). To ensure same total pulse numbers, we applied the following protocols for LFS and HFS, separately. LFS comprised 10 sweeps, each sweep including 100 pulses at 10 Hz, with 10 seconds (secs) intervals between two sweeps and total stimulation lasting 3 mins and 10 secs. HFS comprised 10 sweeps, each sweep including 100 pulses at 100 Hz, with 19 secs intervals between two sweeps and total stimulation lasting 3 mins and 1 sec. For the electrical stimulation studies, HFS was set at 100 Hz, LFS was set at 10 Hz and current intensity was set at 100 μA, for all experiments, unless stated. For ELISA determination of SHH or BDNF levels, we collected all supernatant immediately after electrical stimulation.

### ELISA

Hippocampal neurons were cultured at a density of 2 × 10^5^/cm^2^ in 35 mm dish for 10 days, and incubated with 450 μl normal extracellular solution (in mM: 119 NaCl, 2.5 KCl, 2 CaCl_2_, 2 MgCl_2_, 30 glucose, and 25 Hepes) or Ca^2+^-free extracellular solution (in mM: 119 NaCl, 2.5 KCl, 5 EGTA, 2 MgCl_2_, 30 glucose, and 25 Hepes) in 35 mm dish. Immediately after stimulation, all supernatant was collected and assayed in one well of commercialized ELISA kits coated with antibody against SHH (R&D, USA, Cat. No. MSHH00), or against BDNF (Promega, USA, Cat. No. G7610).

For determination of SHH or BDNF release from slices, hippocampal slices were prepared from SD rats at the age of P20. Animals were anesthetized with 1% pentobarbital sodium, and the brains were rapidly removed. Transverse slices were cut at 400 μm thick on a vibration microtome (Leica VT1000S, Germany) in ice-cold dissection buffer (in mM: 213.26 sucrose, 2.5 KCl, 2 MgSO_4_, 0.5 CaCl_2_, 1.25 NaH_2_PO_4_, 26 NaHCO_3_ and 10 glucose). Before use, the slices were equilibrated in artificial cerebral spinal fluid (aCSF) (in mM: 119 NaCl, 2.5 KCl, 1.3 MgSO_4_, 2.5 CaCl_2_, 1.0 NaH_2_PO_4_, 26.2 NaHCO_3_ and 11 glucose) saturated with 95%O_2_/5%CO_2_ for at least 1 hr. Electrical stimulation was occurred within 450 μl aCSF containing heparin (1 mg/ml, for all experiments, unless stated). Immediately after stimulation, the slices were homogenized and centrifuged at 1,000 g for 10 mins at 4 °C. All supernatant was collected and assayed in one well of commercialized ELISA kits coated with anti-SHH or anti-BDNF antibody. Both were calculated according to the standard curves prepared for each plate using Origin75 software. The standard curves were linear within the range used (0–500 pg/ml), and SHH or BDNF levels in experimental samples were always within the linear range of the standard curves.

### Tirf microscopy and image analysis

The Tirf microscopy study was similar to that reported previously^64^,^65^. An inverted microscopy (Nikon Ti, Japan) equipped with a 60x oil PlanApo Tirf objective (NA 1.49) and a cooled immunoelectron microscopy-CCD camera (Roper Cascade) were used to capture Tirf signals emitted from cultured hippocampal neurons transfected with SHH-pHluorin. For mCherry-positive neurons, axons or dendrites were determined by neuronal morphology. The 488 nm and 594 nm wavelengths were used to stimulate cells. Cells were incubated within normal or Ca^2+^-free extracellular solutions that were warmed at 35 °C. Time-lapse images of pHluorin were acquired with MetaMorph software (Molecular Devices, USA) at 0.2 Hz (acquisition time, 1000 ms). Only mCherry-positive neurons were monitored for fluorescence changes of SHH-pHluorin.

After acquisition, the images were processed for viewing with NIH ImageJ. For identification of mCherry-positive puncta, single frames or stacks of images were used to define a region of interest (ROI). We set a small box as ROI at mCherry-positive neural axons or dendrites to monitor fluorescence changes of SHH-pHluorin. To rule out possible variation in expression levels of SHH-pHluorin and image acquisition conditions among different trials, we presented data as normalized fluorescence changes (ΔF/F_0_, ΔF = F_x_ − F_0_), in which fluorescence changes at a given time (F_x_) were divided by the baseline fluorescence before stimulation (F_0_). Puncta with the amplitude of fluorescence increase ±2 standard deviation from F_0_ were categorized as undergoing a release event. The rest of the events were defined as no fusion. The quantification was done blinded to the treatment of neurons.

### RNAi constructs and infection

Specific sequences of short hair RNA (shRNA) targeting rat SNAP-25, SNAP-23, STX-4, SYT-I, SYT-IV were designed and constructed into the pLentiLox3.7 (pLL3.7) lentiviral vectors separately, all of which have a GFP tag. The lentivirus was packaged and amplified in human embryonic kidney 293T (HEK293T) cells, which were obtained from the Cell Bank of the Chinese Academy of Sciences. The cultured hippocampal neurons were infected at the multiplicities of infection of 5. The shRNA sequences are described below.

Rat shSNAP25-1 forward:

T-(Ggcgaacaactggaacgca)-(TTCAAGAGA)-(tgcgttccagttgttcgcC)-TTTTTTC

Rat shSNAP25-1 reverse:

TCGAGAAAAAA (Ggcgaacaactggaacgca)-(TCTCTTGAA)-(tgcgttccagttgttcgcC)-A

Rat shSNAP25-2 forward:

T-(Gatggccatcagtggtggc)-(TTCAAGAGA)-(gccaccactgatggccatC)-TTTTTTC

Rat shSNAP25-2 reverse:

TCGAGAAAAAA (Gatggccatcagtggtggc)-(TCTCTTGAA)-(gccaccactgatggccatC)-A

Rat shSNAP25-3 forward:

T-(Ggttggatgagcaaggcgaa)-(TTCAAGAGA)-(ttcgccttgctcatccaacC)-TTTTTTC

Rat shSNAP25-3 reverse:

TCGAGAAAAAA (Ggttggatgagcaaggcgaa)-(TCTCTTGAA)-(ttcgccttgctcatccaacC)-A

Rat shSNAP23-1 forward:

T-(Gcacaaggagaatcctggg)-(TTCAAGAGA)-(cccaggattctccttgtgC)-TTTTTTC

Rat shSNAP23-1 reverse:

TCGAGAAAAAA (Gcacaaggagaatcctggg)-(TCTCTTGAA)-(cccaggattctccttgtgC)-A

Rat shSNAP23-2 forward:

T-(Gcggccagcggtggataca)-(TTCAAGAGA)-(tgtatccaccgctggccgC)-TTTTTTC

Rat shSNAP23-2 reverse:

TCGAGAAAAAA (Gcggccagcggtggataca)-(TCTCTTGAA)-(tgtatccaccgctggccgC)-A

Rat shSNAP23-3 forward:

T-(Ggaggcagagaagactttaa)-(TTCAAGAGA)-(ttaaagtcttctctgcctcC)-TTTTTTC

Rat shSNAP23-3 reverse:

TCGAGAAAAAA (Ggaggcagagaagactttaa)-(TCTCTTGAA)-(ttaaagtcttctctgcctcC)-A

Rat shSTX4-1 forward:

T-(Gagaagaacgtggagcgca)-(TTCAAGAGA)-(tgcgctccacgttcttctC)-TTTTTTC

Rat shSTX4-1 reverse:

TCGAGAAAAAA (Gagaagaacgtggagcgca)-(TCTCTTGAA)-(tgcgctccacgttcttctC)-A

Rat shSTX4-2 forward:

T-(Gtgaggtgtttgtgtctaa)-(TTCAAGAGA)-(ttagacacaaacacctcaC)-TTTTTTC

Rat shSTX4-2 reverse:

TCGAGAAAAAA (Gtgaggtgtttgtgtctaa)-(TCTCTTGAA)-(ttagacacaaacacctcaC)-A

Rat shSYTI forward:

T-(Gtgcaagtggtggtaactg)-(TTCAAGAGA)-(cagttaccaccacttgcaC)-TTTTTTC

Rat shSYTI reverse:

TCGAGAAAAAA (Gtgcaagtggtggtaactg)-(TCTCTTGAA)-(cagttaccaccacttgcaC)-A

Rat shSYTIV-1 forward:

T-(Gtgcactcccaacgcagtg)-(TTCAAGAGA)-(cactgcgttgggagtgcaC)-TTTTTTC

Rat shSYTIV-1 reverse:

TCGAGAAAAAA (Gtgcactcccaacgcagtg)-(TCTCTTGAA)-(cactgcgttgggagtgcaC)-A

Rat shSYTIV-2 forward:

T-(Ggccgtctcccctgagagc)-(TTCAAGAGA)-(gctctcaggggagacggcC)-TTTTTTC

Rat shSYTIV-2 reverse:

TCGAGAAAAAA (Ggccgtctcccctgagagc)-(TCTCTTGAA)-(gctctcaggggagacggcC)-A

Nonsense (Non) forward:

T-(Gcgagacaatgcgacacga)-(TTCAAGA)-(tcgtgtcgcattgtctcgC)-TTTTTTC

Nonsense (Non) reverse:

TCGAGAAAAAA (Gcgagacaatgcgacacga)-(TCTCTTGAA)-(tcgtgtcgcattgtctcgC)-A.

### Animals, solutions, reagents and antibodies

SD rats at the ages of E18, P20 or 2 months were purchased from SLAK Laboratory Animal, Shanghai, China. Rats were anesthetized with 10% chloral hydrate before experiments. The use and care of animals were carried out in accordance with approved guideline of the Biomedical Research Ethics Committee and the Institutional Animal Care and Use Committee at the Shanghai Institutes for Biological Science, Chinese Academy of Sciences.

In Tirf microscopy study, Hepes buffer refers to normal extracellular solution (in mM: 119 NaCl, 2.5 KCl, 2 CaCl_2_, 2 MgCl_2_, 30 glucose, and 25 Hepes). The MES (Sigma, USA, Cat. No. M3671) solution was prepared by replacing Hepes with MES at a final pH of 5.2. The NH_4_Cl (Sigma, USA, Cat. No. A9434) solution was prepared by replacing NaCl with NH_4_Cl at a final pH of 7.4. All other components in the solution remained unchanged with Hepes buffer.

Tetrodotoxin (Cat. No. A3109, 1 μM), Poly-D-lysine, heparin (Cat. No. H3149, 1 mg/ml) and nimodipine (Cat. No. N149, 10 μM) were purchased from Sigma. Fetal bovine serum (FBS, Cat. No. 16000044), B-27 (Cat. No. 17504044), glutamax (Cat. No. 35050), DMEM (Cat. No. 11995065), NeuroBasal (Cat. No. 21103-049) and Lipofectamine 2000 (Cat. No. 11668-019) were purchased from Invitrogen, USA. Murine sonic hedgehog (Cat. No. 315-22, 100 ng/ml) was purchased from Pepro Tech, USA. Bafilomycin A1 (Cat. No. 19–148, 1 μM) was purchased from Calbiochem, Germany. All the other reagents were from Sangon, China.

The anti-SHH antibody (Cat. No. 2287 S, 1:1000 dilution for western blots, 1:100 for immunoelectron microscopy) and anti-Gli1 antibody (Cat. No. 2534, 1:1000 dilution for western blots) were purchased from Cell Signaling. The anti-NR2A antibody (Cat. No. M264, 1:1000 dilution for western blots), anti-α-tubulin antibody (Cat. No. T6074, 1:3000 dilution for western blots) and anti-GAPDH antibody (Cat. No. G8795, 1:3000 dilution for western blots) were purchased from Sigma. The anti- axon-specific microtubule associated protein SMI312 antibody (Cat. No. SMI312R, 1: 200 dilution for immunostaining) was purchased from Covance, USA. The anti-microtubule-associated protein 2 (MAP2) antibody (Cat. No. EPR19691, 1: 100 dilution for immunostaining) was purchased from Abcam, UK. The anti-SGII antibody (Cat. No. SC-50290, 1:1000 dilution for western blots, 1:100 for immunoelectron microscopy) was purchased from Santa Cruz. The anti-PSD95 antibody (Cat. No.124011, 1:1000 dilution for western blots, 1:100 dilution for immunostaining), anti-SIN1 antibody (Cat. No.101011, 1:1000 dilution for western blots, 1:100 for immunoelectron microscopy), anti-SNAP25 antibody (Cat. No. 111011, 1:1000 dilution for western blots), anti-SNAP23 antibody (Cat. No. 111203, 1:1000 dilution for western blots), anti-STX4 antibody (Cat. No. 110041, 1:1000 dilution for western blots), anti-SYTI antibody (Cat. No. 105011, 1:1000 dilution for western blots) and anti-SYTIV antibody (Cat. No. 105043, 1:1000 dilution for western blots) were purchased from the Synaptic System, Germany. The HRP-conjugated goat anti-mouse (Cat. No. NA931, 1:3000 dilution for western blots) or anti-rabbit antibody (Cat. No. NA934, 1:3000 dilution for western blots) were purchased from GE Healthcare, UK. The 10-nm or 18-nm gold particle-conjugated anti-rabbit or anti-mouse antibody was purchased from Sigma.

### Statistical analysis

Data were expressed as the means ± s.e.m. The means between two groups were analyzed by paired t-test using Office Excel 2004. Two-way ANOVA for multiple groups’ comparison were calculated by Graphpad Prism. Significance was taken at P < 0.05. Tirf imaging and western blots were repeated for at least three times independently.

## Additional Information

**How to cite this article:** Su, Y. *et al*. High frequency stimulation induces sonic hedgehog release from hippocampal neurons. *Sci. Rep.* **7**, 43865; doi: 10.1038/srep43865 (2017).

**Publisher's note:** Springer Nature remains neutral with regard to jurisdictional claims in published maps and institutional affiliations.

## Supplementary Material

Supplementary Information
