# Supplementary material for: High frequency stimulation induces sonic hedgehog release from hippocampal neurons
Source: Sci Rep. 2017 Mar 6;7:43865. doi: 10.1038/srep43865 (PMC5338313; doi:10.1038/srep43865)
Supplement: Supplementary Information [file srep43865-s1.pdf]

High frequency stimulation induces sonic hedgehog release from hippocampal neurons

Yujuan Su[1], Yuan Yuan[1], Shengjie Feng[1], Shaorong Ma[1] and Yizheng Wang[2*]

[1]Laboratory of Neural Signal Transduction, Institute of Neuroscience and State Key Laboratory of Neuroscience, Shanghai Institutes for Biological Sciences, Chinese Academy of Sciences, University of Chinese Academy of Sciences, Shanghai, China

[2]Laboratory of Neural Signal Transduction, Institute of Neuroscience.

*To whom correspondence should be addressed:

Yizheng Wang, Ph.D.

Institute of Neuroscience,

Chinese Academy of Sciences,

320 Yue-Yang Road,

Shanghai, 200031 P.R. China

E-mail: yzwang@ion.ac.cn.

Supplementary information

Supplementary methods

Whole-cell electrophysiological recordings

Whole-cell current clamp recordings were performed to examine spikes of cultured hippocampal neurons at DIV10 elicited by bath-applied electrical stimulation. Recording pipettes (3.5-4.5 MΩ) were back filled with intracellular solution (in mM: 120 potassium gluconate, 20 KCl, 2 $MgCl_2$, 10 Hepes, 10 EGTA, 2 Na_2ATP, pH 7.3). Data were acquired using MultiClamp 700B (Axon Instruments, USA) and Digidata 1440A, sampled at 10 kHz, and filtered at 2 kHz. Off-line analysis was done by Clampfit 10.0 software (Axon Instruments).

Propidium staining

Immediately after electrical stimulation, hippocampal neurons or slices were firstly washed twice with PBS and fixed in 4% PFA for 20 minutes (mins). For hippocampal slices, the tissues were treated additionally with 0.3% Triton X-100 for 10 mins at RT. The neuronal viability was determined by propidium iodide (PI, Sigma, USA, Cat. No. P4170) and Hochest33342 (Sigma, USA, Cat. No. B2261) staining. The neurons or slices were incubated with PI (2 μg/ml) and Hochest33342 (2 μg/ml) for 10 mins at RT, followed by washing with PBS for three times. The fluorescent signals were examined using an A1R laser-scanning confocal microscopy. The apoptotic rate was determined by the ratio of PI-positive neurons normalized by Hochest33342 from three independent fields per culture well. The data was collected from four independent experiments.

Supplementary figures and figure legends

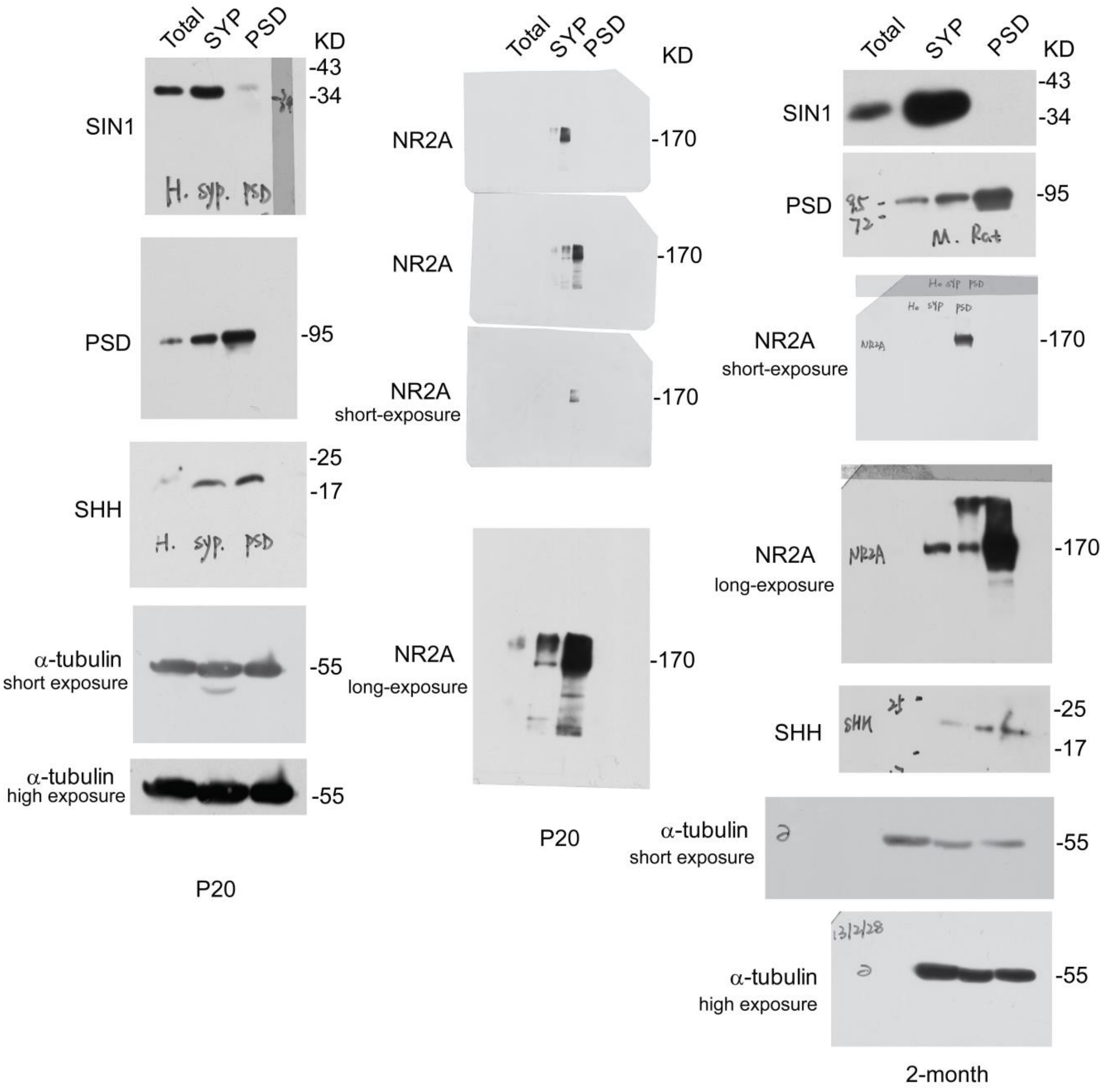

Supplementary Fig. S1. Full-length gels of western blot experiments in Fig. 1a.

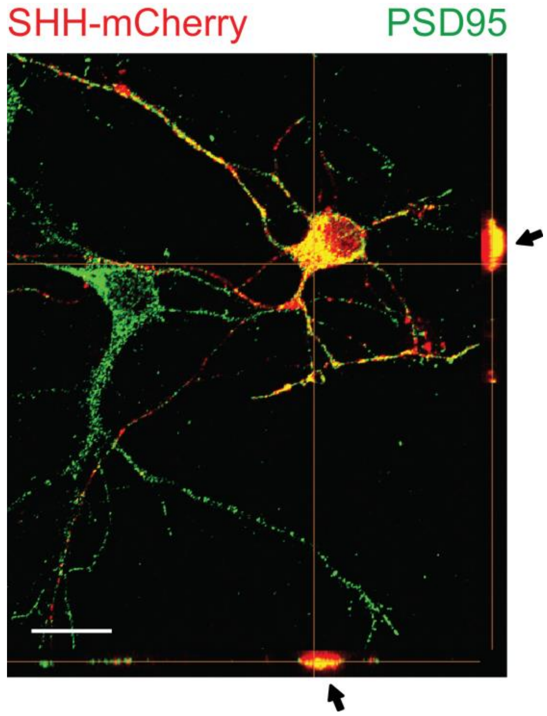

Supplementary Fig. S2. SHH-mCherry expression in the post-synapse of hippocampal neurons. SHH-mCherry expression in the post-synapse of cultured hippocampal neurons through immunostaining with the antibody against PSD95. The co-localization of SHH with PSD95 shown in the orthogonal view are marked by black arrows.

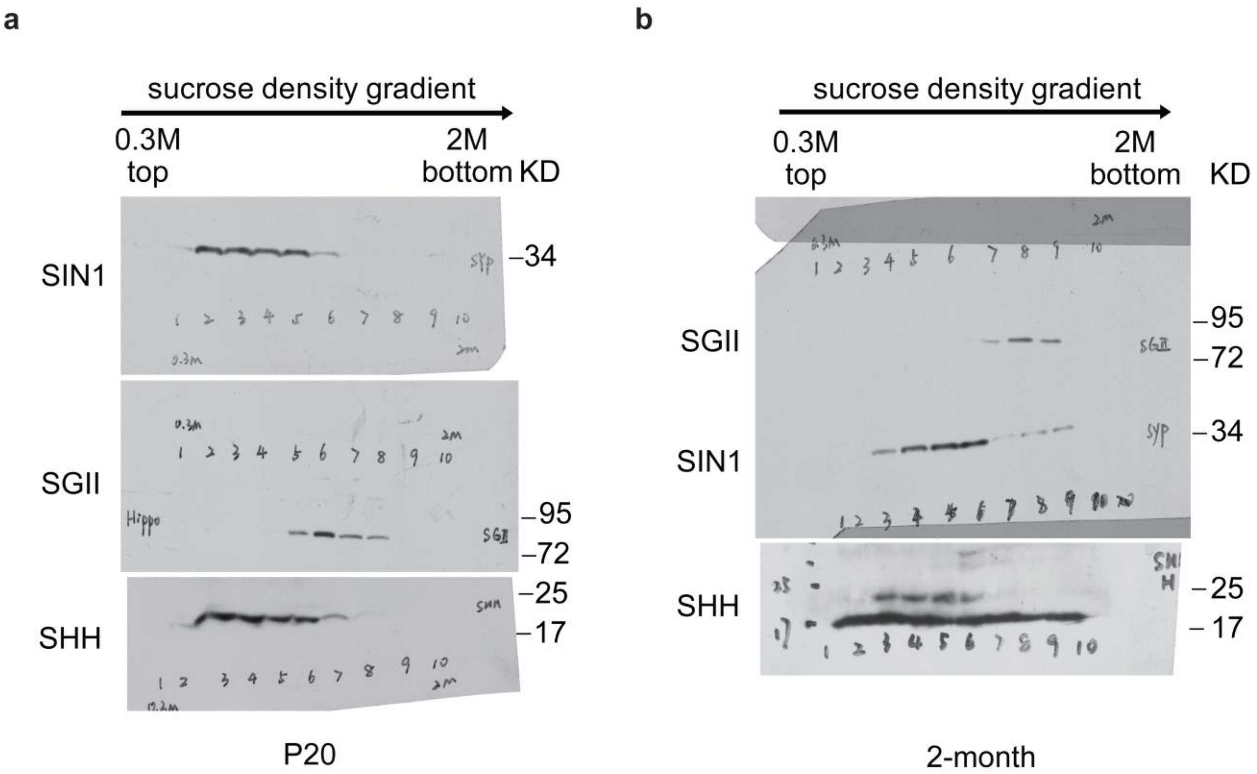

Supplementary Fig. S3. Full-length gels of western blot experiments in Fig. 1d and e. (**a**) Full-length gels of western blots in **Fig. 1d**. (**b**) Full-length gels of western blots in **Fig. 1e**.

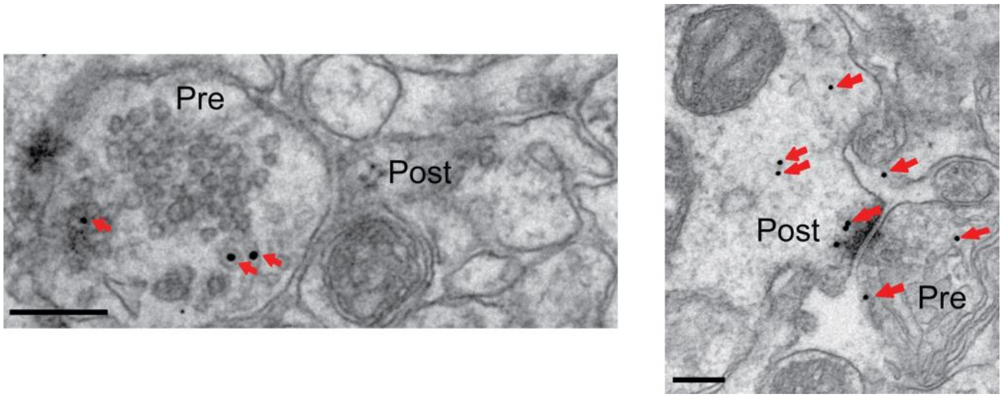

Supplementary Fig. S4. The synaptic localization of SHH assayed by immunogold electron microscopy. Representative electron microscopic images of immunogold labeling by the antibody against SHH (red arrows) showing its synaptic localization in the pre-synaptic terminals (pre) as well as the post-synaptic soma (post) of P20 rat hippocampus. Scale bars: 20 μm.

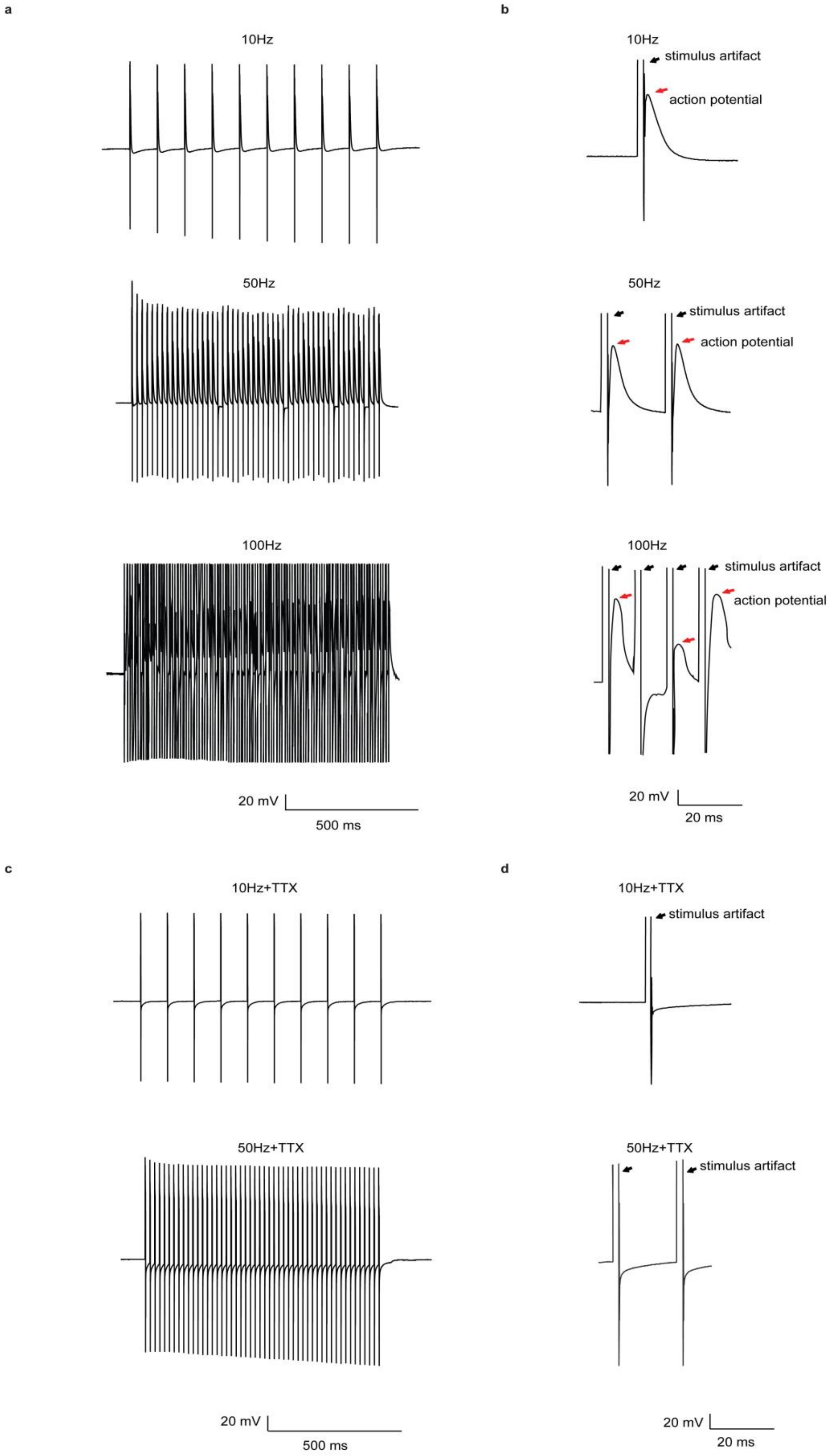
a
10Hz
50Hz
100Hz
20 mV
500 ms
b
10Hz
stimulus artifact
action potential
50Hz
stimulus artifact
action potential
100Hz
stimulus artifact
action potential
20 mV
20 ms
c
10Hz+TTX
50Hz+TTX
20 mV
500 ms
d
10Hz+TTX
stimulus artifact
50Hz+TTX
stimulus artifact
20 mV
20 ms

58

Supplementary Fig. S5. TTX abolished the formation of action potentials in response to bath-applied stimulation. Whole-cell current recording showing action potential formation of hippocampal neurons in response to 10 Hz、50 Hz and 100 Hz stimulation, separately. (**b**) Magnified views of action potentials in (**a**). (**c**) TTX (1 μM) abolished the formation of action potentials in response to 10 Hz and 50 Hz stimulation, separately. (**d**) Magnified views of (**c**). (**b** and **d**) Stimulus artifacts are marked with black arrows, action potentials are marked with red arrows.

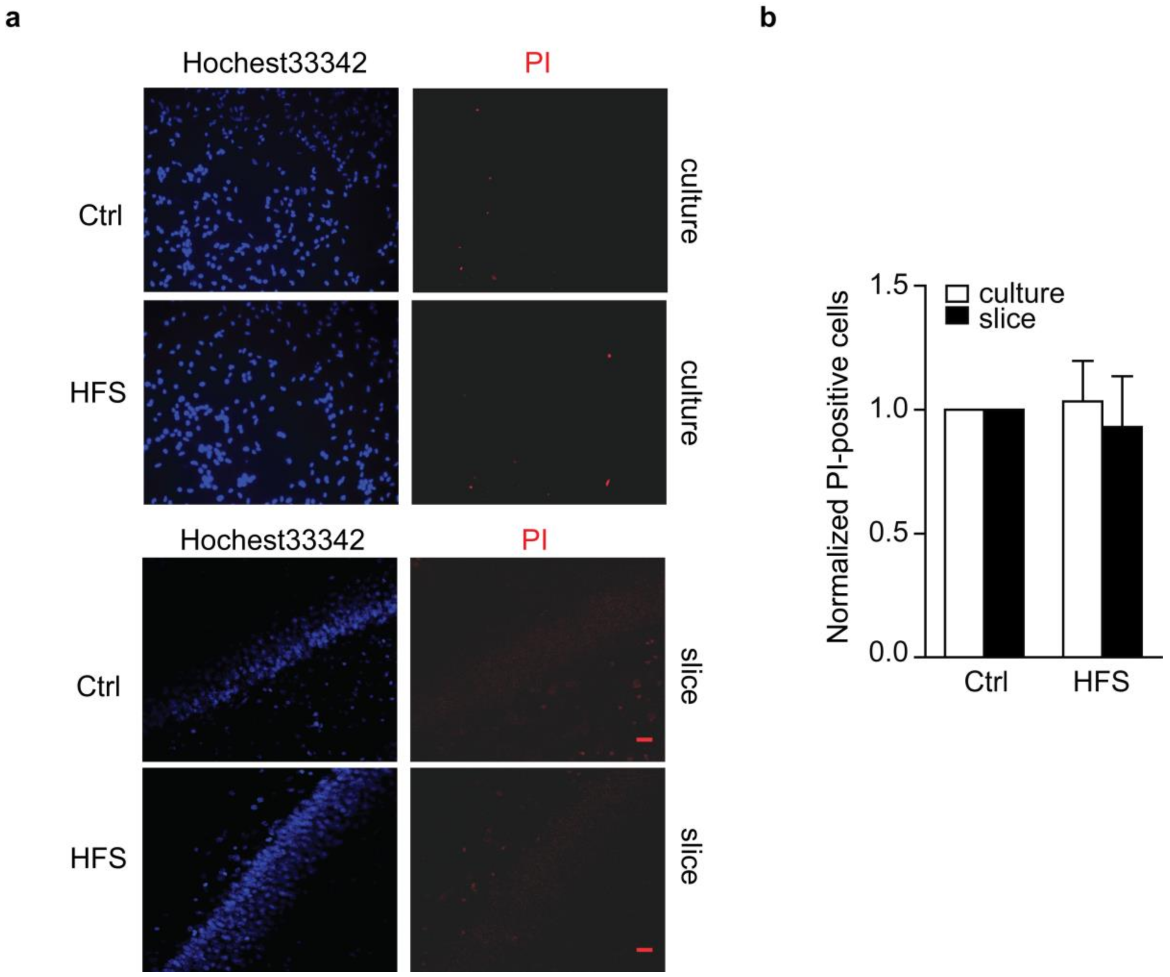

Supplementary Fig. S6. HFS does not induce neuronal damage. (**a**) Representative immunostaining images of cultured hippocampal neurons and acute hippocampal slices stained

with Hochest33342 and PI after Ctrl or HFS (500 μA). Scale bars: 20 μm. (**b**) Statistics for (**a**). N=6, Paired t-test was used. Data were means ± s.e.m.

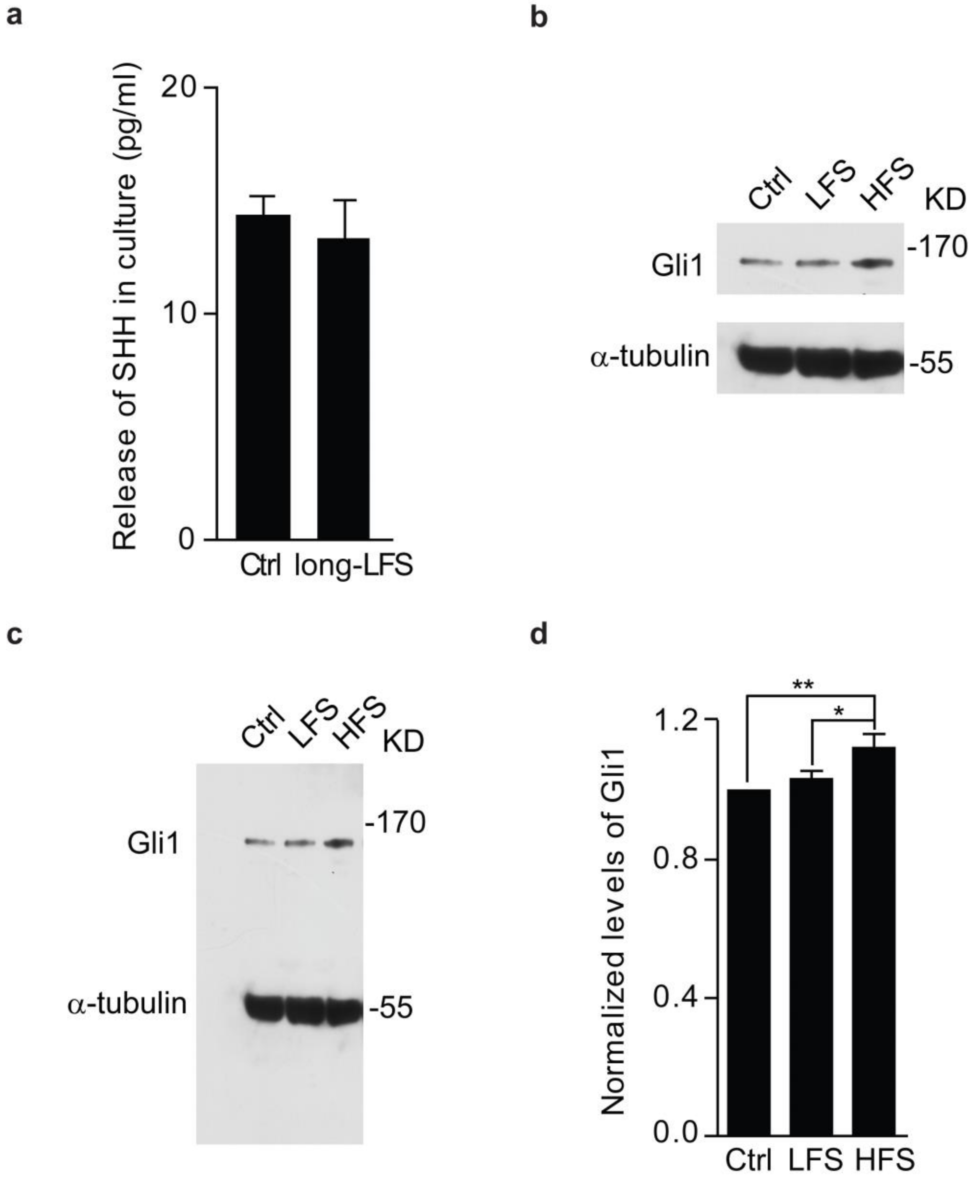

Supplementary Fig. S7. HFS, but not LFS for 30 mins, can induce SHH release from the neurons. (**a**) Levels of SHH in the medium of cultured hippocampal neurons after LFS for 30 mins (long-LFS) (N=8). (**b**) Western blots of hippocampal neurons showing Gli1 up-regulation 24 hrs after HFS. (**c**) Full-length gels of western blots in (**b**). (**d**) Quantification of Gli1 protein levels 24 hrs after Ctrl, LFS or HFS. N=7, P=0.0032 for HFS vs. Ctrl, P=0.033 for HFS vs. LFS.

Control (Ctrl): no stimulation. α-tubulin: loading control. HFS: 100 Hz, LFS: 10 Hz, current intensity: 100 μA, for all experiments, unless stated. Paired t-test was applied. Data were means ± s.e.m. *P<0.05, **P<0.01.

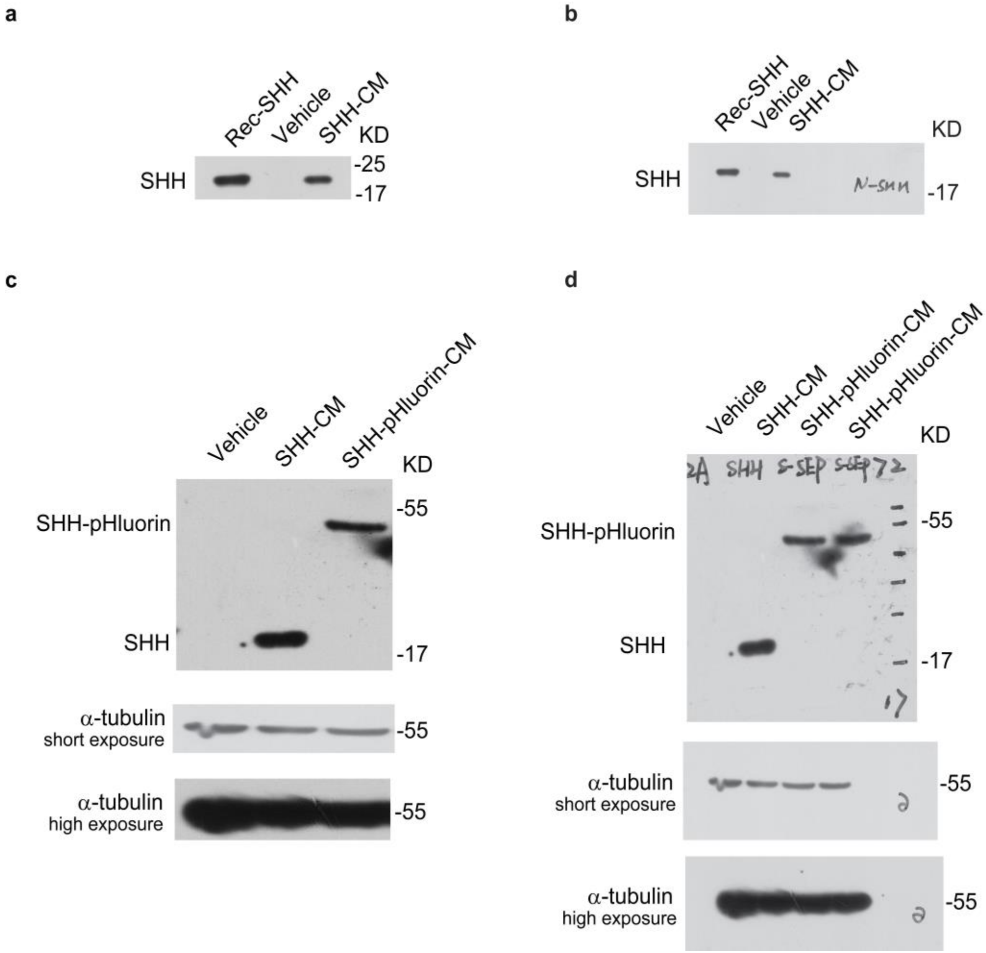

Supplementary Fig. S8. Confirmation of expression and release of SHH and SHH-pHluorin. (**a**) Western blots for SHH in the medium of HEK293 cells transfected with empty vector (vehicle) or C terminal-modified SHH signal peptide (SHH-CM) for 48 hrs using the antibody against SHH. Recombinant sonic hedgehog (Rec-SHH): positive control, which is the commercialized N-terminal signal peptide of SHH, well-decorated and ready to release. (**b**) The full-length gels of western blots in (**a**). (**c**) Western blots for SHH in the medium of HEK293 cells transfected with empty vector (vehicle), SHH-CM or SHH-pHluorin (SHH-pHluorin-CM)

for 48 hrs. α-tubulin: loading control. (**d**) The full-length gels of western blots in (**c**). We load SHH-pHluorin (SHH-pHluorin-CM) twice in order to verify the result.

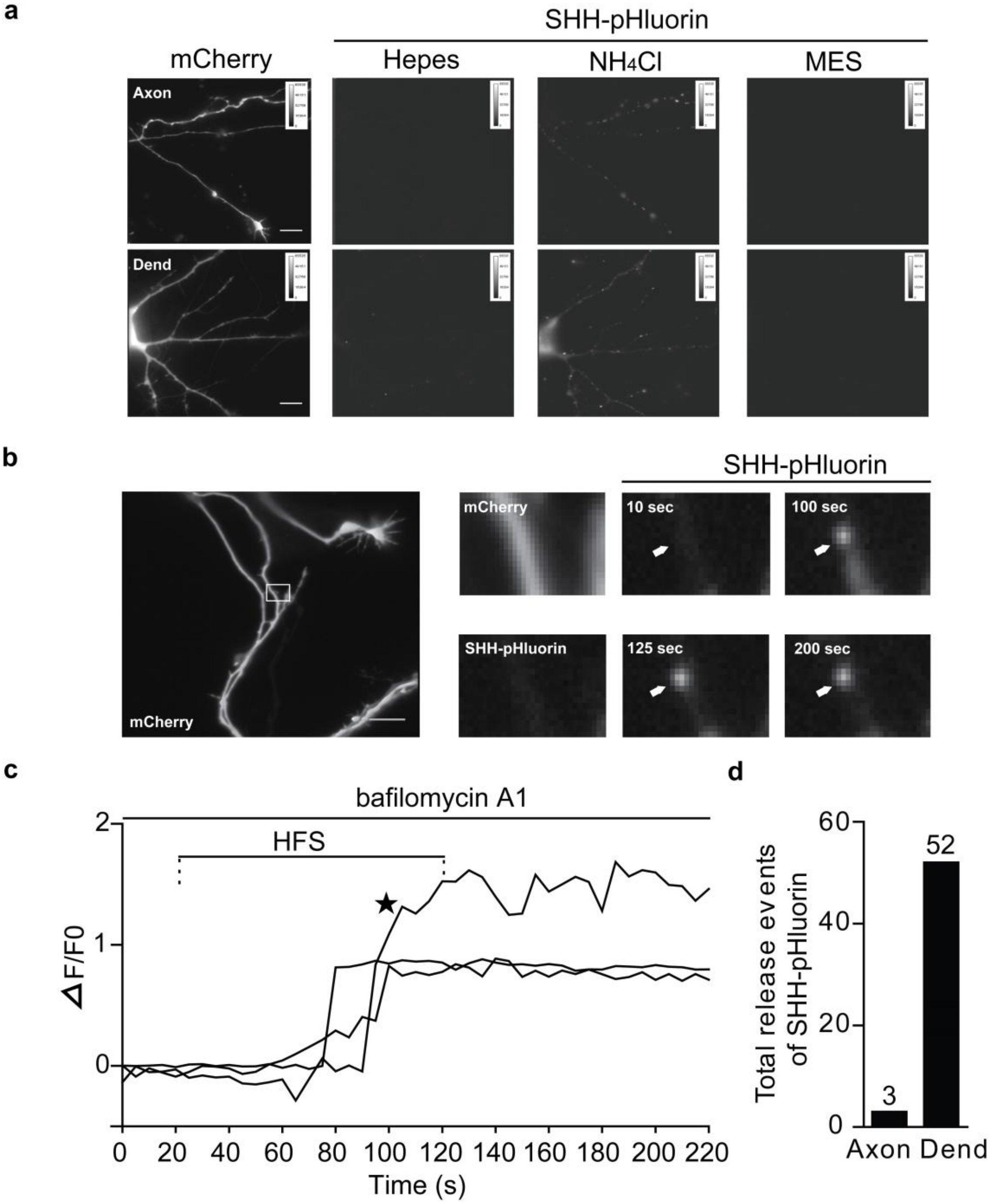

Supplementary Fig. S9. HFS can induce SHH-pHluorin release from hippocampal neurons. (**a**) Representative images of hippocampal neuron transfected with SHH-pHluorin and mCherry at basal (F_0) or with sequential Hepes, NH_4Cl and MES perfusion in axon (Axon) or dendrites

(Dend). (**b**) (Left) Representative images (mCherry) of the neurons transfected with SHH-pHluorin and mCherry in the presence of bafilomycin A1. (Right) Magnified views of SHH-pHluorin punctum in indicated boxed area (Left) at basal condition before stimulation (10 sec, Upper, Middle), or at indicated seconds during (100 sec, Upper, Right), or after (125 sec, 200 sec, Down) stimulation. HFS was applied from 20 to 120 secs. Scale bars in (**a** and **b**): 10 μm. (**c**) Representative fluorescence changes of SHH-pHluorin at dendrites in the presence of bafilomycin A1. The asterisk marks trace showing fluorescence changes of arrow-indicated punctum in (**b**, Right). $\triangle F/F_0$, $\triangle F=F_x-F_0$, F_0, basal fluorescence intensity before stimulation, F_x, fluorescence intensities at indicated time x. (**d**) Total release events of SHH-pHluorin from the Axon and Dend of the neurons after HFS. Numbers above columns are release events in thirty observation fields from at least three independently experiments.

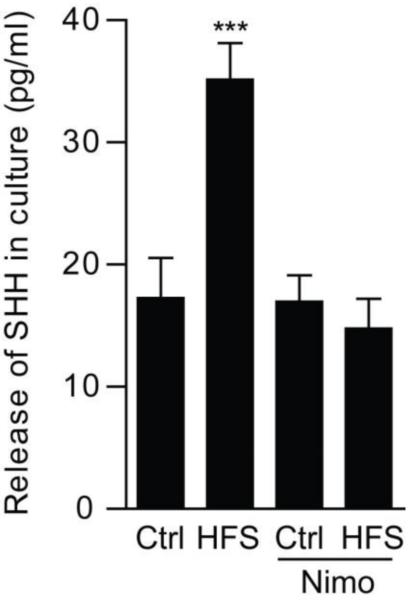

Supplementary Fig. S10. Nimo reduced the HFS-induced SHH release from the cultured hippocampal neurons. Levels of SHH after HFS in the medium of cultured hippocampal neurons with or without Nimo ($F(1,10)=28.13$, $P=0.0003$ for HFS vs. Ctrl without Nimo). Two-way ANOVA was used. Data were means ± s.e.m. ***$P<0.001$.

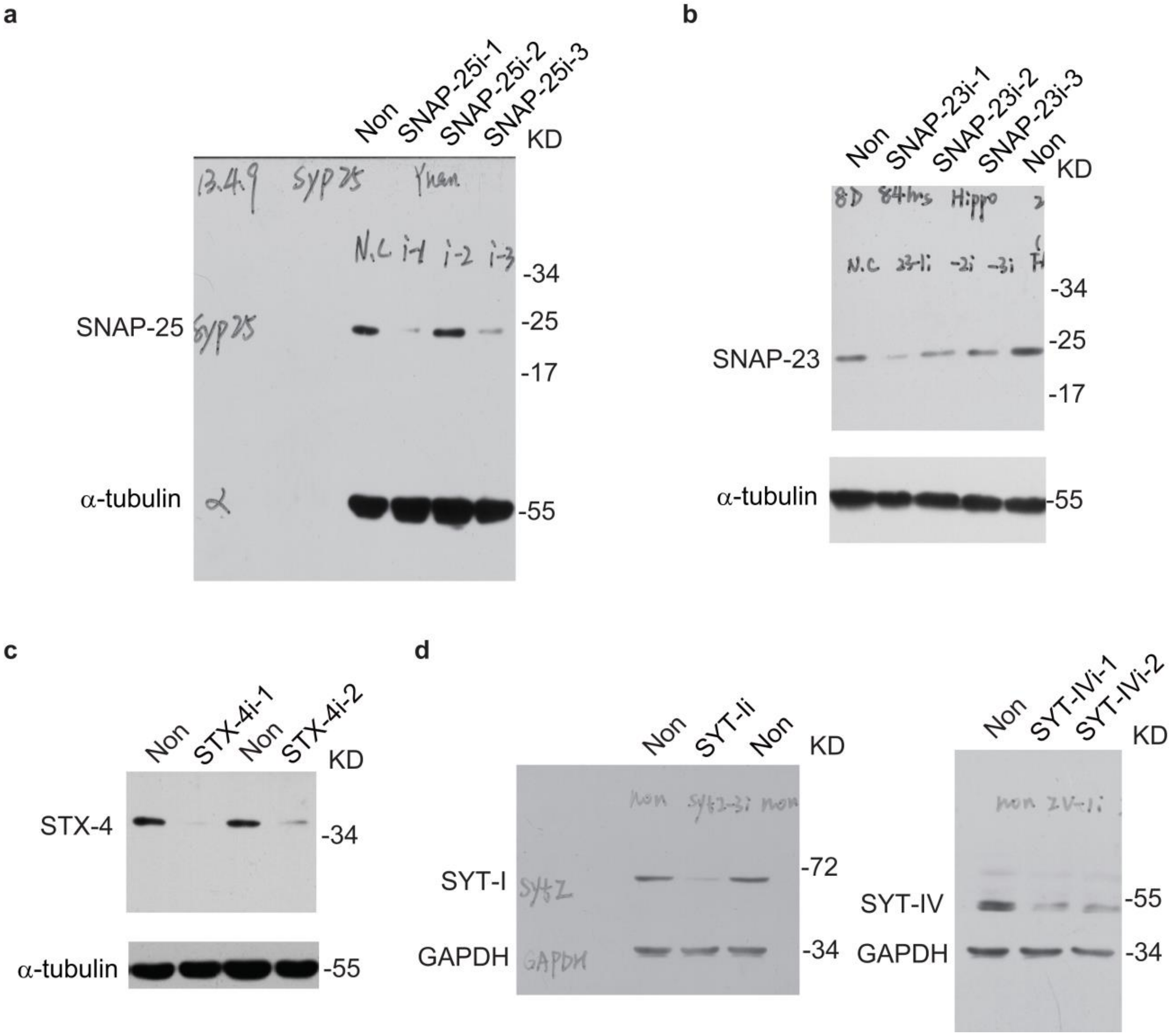

Supplementary Fig. S11. Full-length gels of western blot experiments in Fig.4.

(**a-d**) Full-length gels of western blots in **Fig. 4d-g**.

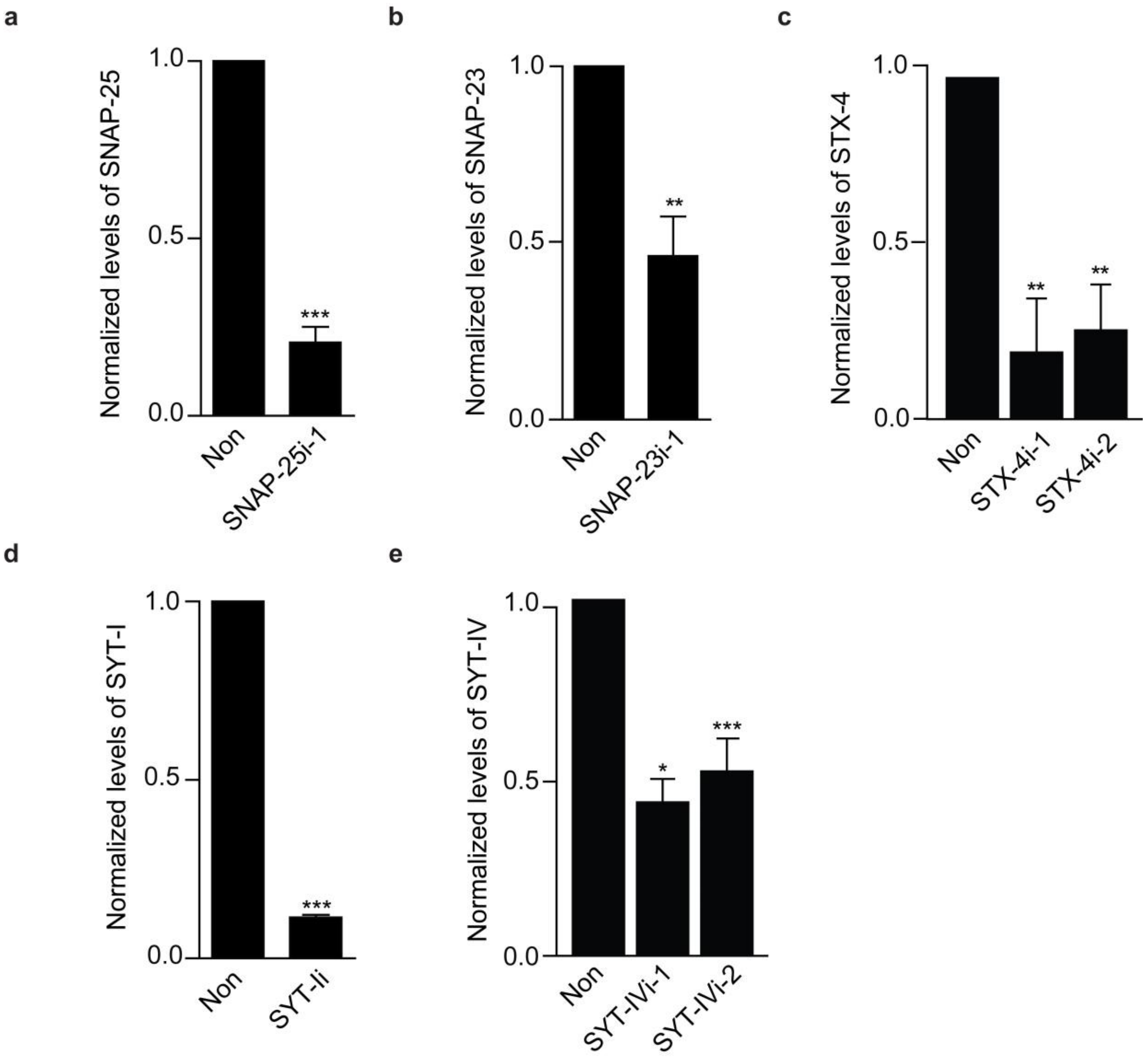

Supplementary Fig. S12. Effects of RNAi on the expression of proteins involved in vesicle release. (**a-e**) Statistics of the indicated protein levels in the neurons transfected with shRNA against SNAP-25 (SNAP-25i-1, **a**, N=3, P=3.46E-05 vs. Non), SNAP-23 (SNAP-23i-1, **b**, N=4, P=0.0027 vs. Non), STX-4 (STX-4i-1, STX-4i-2, **c**, N=3, P=0.006 for STX-4i-1 vs. Non, P=0.005 for STX-4i-2 vs. Non), SYT-I (SYT-Ii, **d**, N=3, P=8.13E-05 vs. Non) and SYT-IV (SYT-IVi-1, SYT-IVi-2, **e**, N=4, P=0.011 for SYT-IVi-1 vs. Non, P=4.15E-05 for SYT-IVi-2 vs. Non) shown in **Fig. 4c-f**. Nonsense RNAi (Non): negative control. Paired t-test was used. Data were means ± s.e.m. *$P<0.05$, **$P<0.01$, ***$P<0.001$.

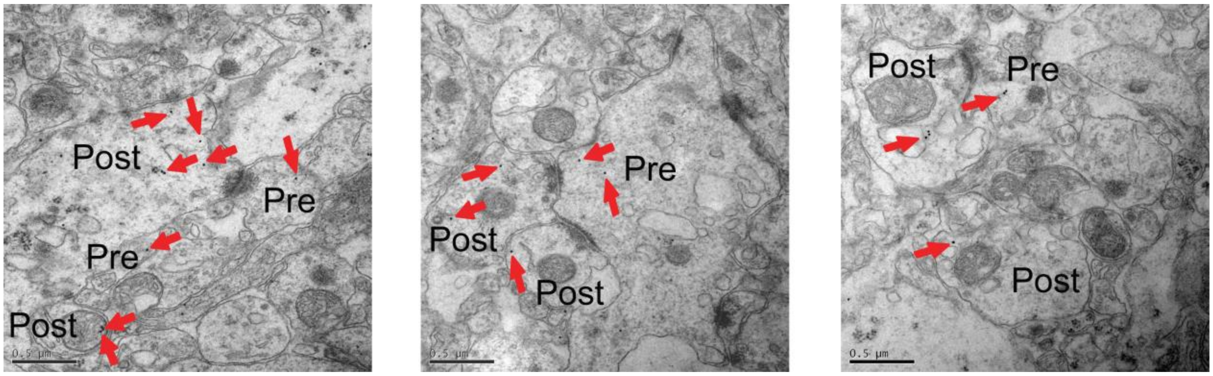

Supplementary Fig. S13. Electron microscopic images showing synaptic vesicle localization in the synapse. Electron microscopic images of immunogold labeling showing localization of synaptic vesicles (labeled with the antibody against synaptophysin1, SIN1, red arrows) in both pre-synaptic terminals (pre) and post-synaptic soma (post). Scale bars: 0.5 μm.

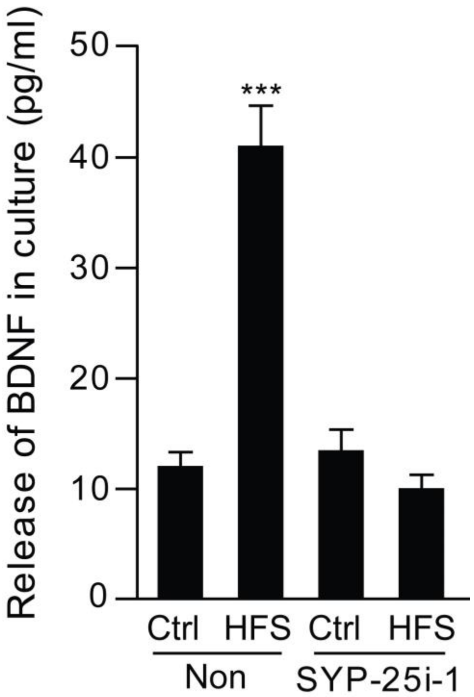

Supplementary Fig. S14. SNAP-25 is necessary for HFS-induced BDNF release. Levels of BDNF after HFS in the medium of cultured hippocampal neurons transfected with nonsense RNAi (Non) or RNAi against SNAP-25 (SNAP25-i-1). N=5, $F(1,8)= 48.84$, $P=0.0001$ for HFS vs. Ctrl). Two-way ANOVA was used. Data were means ± s.e.m. ***$P<0.001$.
